# Multifactorial lifestyle interventions in rheumatoid arthritis: a systematic literature review

**DOI:** 10.1016/j.ero.2026.100254

**Published:** 2026-10-08

**Authors:** Megan Curran, Kevin Durkan, Afroja Ahmed, Kate McCann, Norelee Kennedy, Audrey Tierney

**Affiliations:** a School of Allied Health, University of Limerick, Limerick, Ireland; b Centre for Implementation Research, Health Research Institute, University of Limerick, Limerick, Ireland; c Food, Diet and Nutrition Research Group, Health Research Institute, University of Limerick, Limerick, Ireland; d Department of Sport, Exercise and Nutrition, Atlantic Technological University, Galway, Ireland; e Emdoc Health, Sandyford, Dublin, Ireland

## Abstract

**Objectives:**

Although research investigating the role of lifestyle factors in rheumatoid arthritis (RA) management is growing, evidence regarding multifactorial lifestyle interventions remains limited. The aim of this systematic review is to examine the evidence on multifactorial lifestyle interventions for adults with RA and their effects on disease activity, inflammatory markers, fatigue, physical function, pain, and quality of life.

**Methods:**

Five databases (PubMed, SPORTdiscus, Embase, Medline, and CINAHL) were searched in February 2024 and June 2026 to retrieve clinical trials investigating lifestyle interventions in adults with RA on disease activity, inflammatory markers, pain, function, fatigue, and quality of life. Risk of bias was assessed using the Cochrane Risk of Bias tool. Key study data were extracted, and because of study heterogeneity, results were synthesised narratively.

**Results:**

Of 2992 screened records, 12 studies met the inclusion criteria, including a total of 660 participants. Some studies demonstrated significant and clinically important improvements in outcome measures; however, findings varied across studies and outcome measures. Two studies demonstrated potential advantages of multifactorial lifestyle interventions over single-component interventions.

**Conclusions:**

Multifactorial lifestyle interventions may improve disease activity and other core outcome measures in adults. However, findings were inconsistent across studies, with interpretation limited by varying baseline characteristics, small sample sizes, methodological limitations, and low-certainty evidence. Larger, high-quality trials are needed to confirm these findings and evaluate the effectiveness, feasibility, acceptability, and implementation of multifactorial lifestyle interventions in RA management.

**WHAT IS ALREADY KNOWN ON THIS TOPIC**

 

• There is growing evidence supporting the role of individual lifestyle factors in rheumatoid arthritis (RA) management. However, reviews synthesising the effects of multifactorial lifestyle interventions in RA remain limited.

**WHAT THIS STUDY ADDS**

 

• This review provides the first comprehensive synthesis of multifactorial lifestyle interventions, including diet, physical activity, sleep, mental well-being, stress, and substance use, with some studies providing evidence of improvements in disease activity and Outcome Measures in Rheumatology core outcomes, while overall demonstrating that the available evidence remains limited, heterogeneous, and of low quality.

**HOW THIS STUDY MIGHT AFFECT RESEARCH, PRACTICE OR POLICY**

 

• By establishing the current evidence base, this review identifies priorities for the development and evaluation of future multifactorial lifestyle interventions in RA. The findings suggest that multifactorial lifestyle interventions may improve disease activity and other core outcome measures; however, further high-quality research is needed to determine their effectiveness, feasibility, acceptability, and implementation in routine RA management.

Alt-text: Unlabelled box dummy alt text

## INTRODUCTION

Rheumatoid arthritis (RA) is a chronic autoimmune disease characterised by inflammation of the joints, causing pain, stiffness, swelling, weakness, and fatigue [1]. RA affects an estimated 18 million people globally and disproportionately affects women [2,3]. The pathophysiology of RA involves complex inflammatory processes that contribute to inflammation and subsequent joint damage, including an overproduction of cytokines such as tumour necrosis factor (TNF) [4,5]. The exact aetiology of RA is unknown, but it is considered multifactorial, involving genetic susceptibility, environmental triggers, and modifiable lifestyle factors [6]. Tobacco use is the most established risk factor, with emerging evidence also implicating poor diet quality, obesity, and physical inactivity [6]. RA is commonly associated with comorbidities, including cardiovascular disease (CVD), gastrointestinal disorders, infections (including tuberculosis), and osteoporosis [[7], [8], [9]]. The burden of RA accompanied by comorbidities can adversely affect physical and mental health, quality of life, and disability [8,10].

Although pharmacological treatments are commonly used to manage RA, some patients experience suboptimal treatment responses or adverse effects [11,12]. A recent systematic review also identified smoking, obesity, and depression as factors significantly associated with the occurrence of difficult-to-treat RA, highlighting the role of lifestyle factors in RA management [13]. Consequently, there is growing evidence supporting the role of lifestyle medicine as an adjunct to pharmacological management [14]. Lifestyle medicine is an evidence-based healthcare approach utilising 6 key lifestyle pillars, including nutrition, physical activity, sleep, social connection, stress management, and minimising risky behaviours to prevent, treat, and manage chronic conditions [15,16]. Lifestyle medicine interventions have demonstrated improvements in cardiovascular risk markers, glycaemic control, and quality of life in chronic diseases such as CVD and diabetes [17,18].

Research is increasingly exploring how lifestyle medicine factors influence RA pathophysiology and disease severity. Several lifestyle factors may act on similar inflammatory processes that may promote or suppress inflammation and subsequently influence RA disease activity and symptoms [[19], [20], [21], [22]]. Dietary patterns including the Mediterranean diet, characterised by a high intake of fruits, vegetables, whole grains, and olive oil, moderate consumption of dairy products and poultry, and limited intake of confectionery and red meat, are high in polyphenols and antioxidants, which have been associated with anti-inflammatory effects [19]. This dietary pattern is recognised for its potential to improve pain, physical function, and cardiovascular health in people with RA [23]. Regular physical activity may promote anti-inflammatory processes and improve physical function, fatigue, quality of life, and pain in people with RA [20,24]. Smoking has been shown to impact the immune system and induce a systemic proinflammatory state and is associated with greater disease severity and an increased risk of RA complications, whereas alcohol consumption has been linked to increased disease incidence and flare-ups [21,[25], [26], [27]]. Furthermore, research has suggested that psychological stress may activate physiological stress pathways, influence immune functioning, and increase inflammation [28]. In addition, poor sleep and sleep deficiency may alter inflammatory mediators and are associated with increased pain, fatigue, anxiety, and depression in RA [22,29].

Given the cumulative evidence that individual lifestyle factors may modulate inflammatory processes, it is plausible that multifactorial lifestyle interventions may be more effective at optimising health outcomes by exerting synergistic effects through targeting multiple related pathways in RA pathophysiology, collectively reducing inflammatory burden compared with interventions targeting a single lifestyle factor.

Multifactorial lifestyle interventions target ≥2 lifestyle factors, such as nutrition, physical activity, or risk-related behaviours, using strategies including advice, education, and counselling to support sustained lifestyle changes [30,31]. These interventions are typically delivered alongside pharmacological management to improve health outcomes and quality of life [32]. Multifactorial lifestyle approaches have been investigated in other chronic conditions, with evidence suggesting improvements in health outcomes, reduced mortality risk, and a more realistic approach to behaviour change by addressing the complex interplay of lifestyle factors [17,18,33].

Despite the evidence, reviews investigating multifactorial lifestyle interventions in RA are limited. One previous systematic review and meta-analysis evaluating dietary and exercise interventions in inflammatory arthritis, including RA, reported significant improvements in physical function; however, substantial study heterogeneity limited the strength of the conclusion [34]. In addition, this review did not investigate lifestyle factors, including sleep, substance use, and stress, which may also affect RA outcomes.

Thus, the aim of this systematic review is to examine the evidence on multifactorial lifestyle interventions and their effects on core outcomes (disease activity, inflammatory markers, fatigue, function, and pain) and quality of life in adults with RA.

## METHODS

### Study design

This review adheres to the Preferred Reporting Items for Systematic Reviews and Meta-Analyses (PRISMA) guidelines [35] and was registered on The International Prospective Register of Systematic Reviews (PROSPERO) (May 27, 2024; CRD42024551640) after the initial literature search. The PROSPERO record was subsequently updated to reflect amendments made after registration, including an updated literature search conducted in 2026, an extension of the publication date ranges from 2014 to 2026, and the addition of a third reviewer to the study screening, data extraction, and quality appraisal processes.

### Search and selection process

A literature search was conducted across 5 databases: PubMed, SPORTDiscus, Embase, Medline, and CINAHL between January and February 2024, with an updated literature search conducted in June 2026. Two independent reviewers (MC and KD) conducted the initial search and screening, whereas a third reviewer (AA) performed the updated search in 2026. The Population, Intervention, Comparator, Outcome framework was used to define eligibility criteria (Table 1). Studies published in English, between 2014 and 2026, evaluating multifactorial lifestyle interventions (targeting ≥2 lifestyle factors), including psychological, behavioural, or self-management interventions, in adults with a definite diagnosis of RA were included. Interventions delivered alongside pharmacological treatment were also eligible. The initial timeframe of 2014 was chosen to identify relevant studies from the 10 years before the commencement of the review. To ensure the most accurate reflection of current evidence before publication, the timeframe was extended to 2026, reflected by an updated literature search. The full search strategy, including keywords, Boolean operators, and truncations, is provided in Appendix A.

**Table 1:** Inclusion and exclusion criteria of studies

	Population	Intervention	Control/comparator	Outcome
Inclusion criteria	- Human studies published in English - Adult population (over 18 y) - Definite diagnosis of RA	- Clinical trials that assess the effect of ≥2 lifestyle factors, including follow-up studies of these trials - Lifestyle factors include dietary approaches, physical activity, stress management, sleep, psychological well-being, risky behaviours, including smoking cessation and alcohol consumption	Participants receiving a placebo, an individual lifestyle intervention, standard care with or without pharmacological management or a comparison of 2 multifactorial lifestyle interventions	RA outcomes, including disease activity, pain, fatigue, physical function, quality of life, and inflammatory markers
	Population	Intervention	Control/comparator	Outcome
Exclusion criteria	- Nonhuman studies - Children population - Participants who do not have RA. This includes participants at risk of RA but do not have an RA diagnosis, or participants with other forms of inflammatory arthritis where RA-specific data could not be extracted	- Studies that assess one lifestyle factor - Studies comparing one lifestyle factor to another lifestyle factor - Studies that do not have a clinical trial study design		- Studies that assessed the risk of RA development - Studies focusing on comorbidity outcomes such as CVD or osteoporosis

CVD, cardiovascular disease; RA, rheumatoid arthritis.

### Title and abstract screening

Studies retrieved from database searches were imported into Covidence. Titles and abstracts were independently screened by 2 researchers (MC and KD), with disagreements resolved by a third researcher (AT). Full texts were assessed against the prespecified eligibility criteria, and reference lists of included studies were screened to identify additional relevant articles.

### Data extraction

Data were independently extracted by 3 reviewers (MC, KD, and AA) using a standardised data extraction table. Studies identified from the initial literature search were divided between 2 reviewers (MC and KD), who independently extracted data. After the updated literature search in 2026, the newly identified eligible studies were divided between MC and AA, who independently extracted the data. All data extractions were independently verified by a second reviewer to ensure accuracy. Extracted data included study author, study design, study population, intervention characteristics, comparator, outcome measures, and quantitative and descriptive findings.

### Study quality assessment

Quality assessment was completed independently by researchers (MC and KD) with disagreements resolved with a third reviewer (AT). The revised Cochrane Risk of Bias tool (RoB-2) was used to assess risk of bias in randomised trials [36]. The RoB-2 tool evaluates a study’s internal validity across 5 domains: (1) risk from the randomisation process; (2) deviations from the intended interventions; (3) missing outcome data; (4) outcome measurement; and (5) selection of reported results. Each domain is rated as low risk, high risk, or some concern, resulting in an overall risk of bias score [36].

### Outcome measures

The primary outcome measure being assessed was disease activity. Disease activity was primarily assessed using the validated 28-joint Disease Activity Score (DAS28), calculated from tender and swollen joint counts, C-reactive protein (CRP) or erythrocyte sedimentation rate (ESR), and a patient’s global health visual analogue scale (VAS) [37]. DAS28 scores <2.6 were considered consistent with remission, 2.6 to <3.2 indicated low disease activity, 3.2 to ≤5.1 indicated moderate disease activity, and >5.1 indicated high disease activity [37]. Secondary outcome measures assessed were derived from the Outcome Measures in Rheumatology (OMERACT) approach, including inflammatory markers (CRP and ESR), pain, fatigue, physical function, and quality of life [38]. Statistical significance of changes in outcome measures was assessed using *P* values, with *P* < .05 considered statistically significant. The minimum clinically important difference (MCID) reported in the RA literature for each outcome measure was used to assess the clinical relevance of observed changes, with changes exceeding the MCID considered clinically important [39].

### Data synthesis

Because of heterogeneity in intervention characteristics and outcome measures, a descriptive synthesis was undertaken, and no meta-analysis, formal sensitivity analyses, assessment of reporting bias, or statistical exploration of heterogeneity were conducted. Studies were grouped according to intervention focus within the summary table and synthesised narratively: (1) interventions targeting diet and physical activity, and (2) comprehensive lifestyle interventions targeting multiple lifestyle factors. Studies reporting quantitative data for key outcomes were summarised in quantitative tables, including baseline and postintervention values for intervention and control groups. Where reported, within-group changes for both intervention and control groups were presented in the text to facilitate comparisons, alongside between-group differences and associated *P* values. Where between-group differences were not reported, this was indicated in the text.

### Grading the evidence

Two researchers (MC and AA) assessed the certainty of evidence across outcome measures using the Grading of Recommendations Assessment, Development and Evaluation (GRADE) approach [40]. Evidence from randomised controlled trials (RCTs) was initially rated as high certainty and downgraded based on risk of bias, inconsistency, indirectness, imprecision, and publication bias [40,41]. Because of study heterogeneity, the certainty of evidence was reported narratively.

## RESULTS

### Results of searching and selection procedure

Results of the database search and study selection are presented in the PRISMA flow diagram (Fig 1). A total of 3628 records were identified across 5 databases, with 636 duplicates removed. Sixty full-text articles were assessed for eligibility, with 12 publications representing 9 unique trials included in this review.

**Figure 1:**
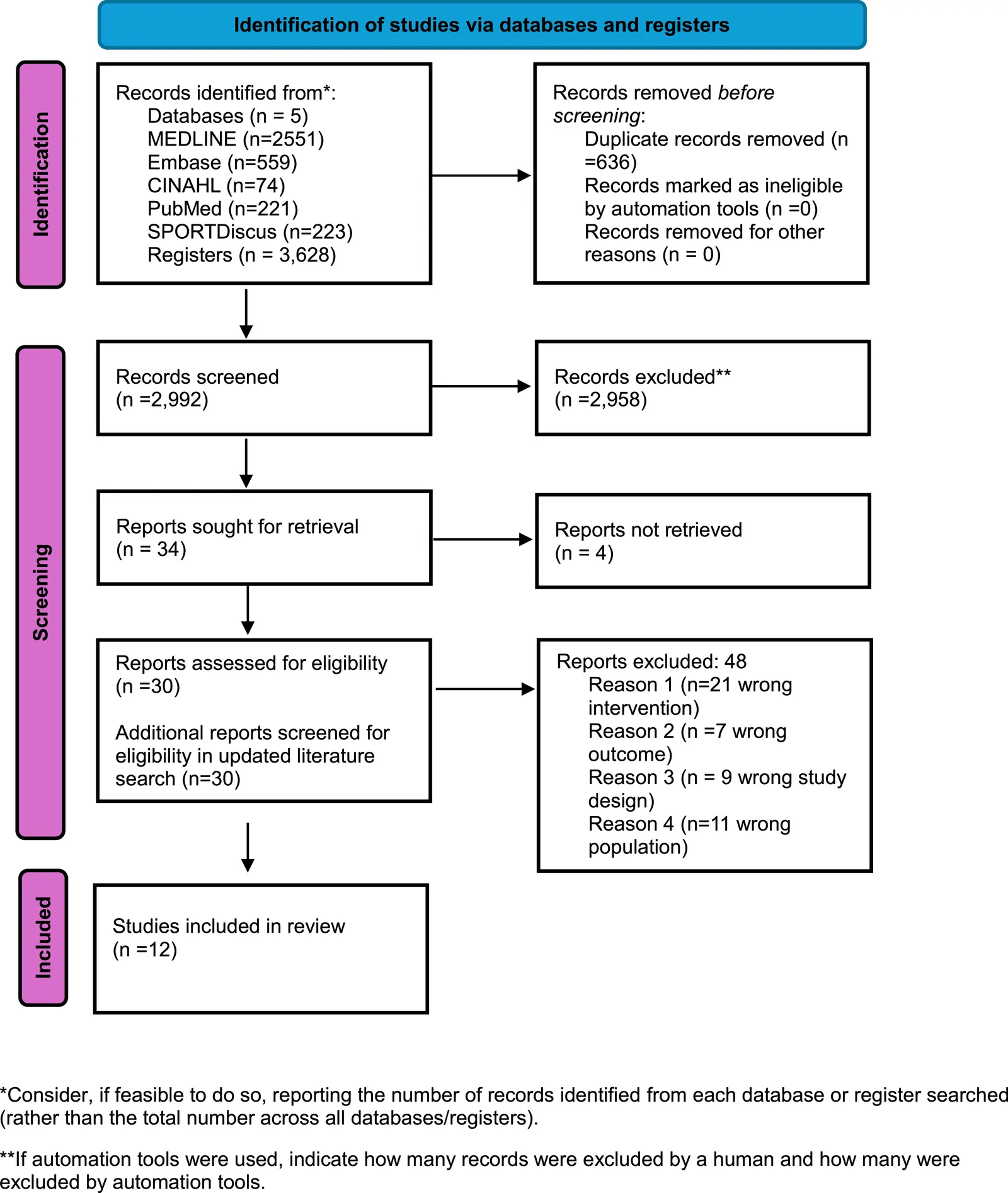
PRISMA flow diagram.

### Characteristics of included studies

Of the 9 unique trials, all 9 were RCTs [[42], [43], [44], [45], [46], [47], [48], [49], [50]], andthe 3 other publications reported follow-up data from included RCTs, with 1 reporting 24-month outcomes [51] and 2 reporting 12- and 24-month outcomes from the same trial [52,53]. Three trials were conducted in Europe [[45], [46], [47]], 1 in Mexico [44], 1 in the USA [43], 3 in Asia [[48], [49], [50]], and 1 in Africa [42]. A total of 660 participants were included in this review, with disease duration ranging from 6 months to 17.5 years. Five trials investigated combined diet and exercise interventions. Three evaluated Mediterranean diet and exercise interventions, including the The Mediterranean DiEt In Rheumatoid Arthritis (MADEIRA)trial [42,44,46]. Other interventions evaluated combined exercise with nutritional guidance [49] or a hypocaloric diet (Supervised Weight loss and Exercise Training [SWET] trial) [43]. A 2-year follow-up of the SWET trial was also included, in which control participants received the intervention materials [51]. Two trials incorporated diet and exercise alongside additional lifestyle factors. The Plants for Joints (PFJ) trial and its 1-year and 2-year follow-up studies evaluated an intervention comprising a plant-based diet, physical activity, stress management, and sleep advice [47,52,53]. One study investigated a combined Mediterranean diet, physical activity, mental health support, and smoking cessation intervention [45]. An additional study investigated stress management, sleep hygiene, physical activity, and social participation [50]. In addition, 1 study compared a cognitive behavioural intervention incorporating stress management and sleep advice with a mindfulness-based stress reduction intervention incorporating stress management and exercise advice [48]. Four studies included sleep, stress management, mental well-being, or social participation components [45,47,48,50], and 1 included smoking cessation [45]. Alcohol consumption was not assessed. Table 2, Table 3 summarise intervention characteristics, whereas Table 4 summarises quantitative results for key clinical trial outcomes.

**Table 2:** Characteristics and main findings of studies assessing diet and exercise interventions

Study	Population	Intervention		Outcome measures	Key findings
Abdel-Aal et al [42] (Egypt) 12-mo double blind parallel RCT	n = 60 female participants (mean age 39.5 ± 4.4 y). Duration of disease = 3.89 y	Intervention group (n = 30) participating in Mediterranean Diet and supervised exercise programme (aerobic and resistance exercises) vs control group (exercise only)		Pain-VAS CRP-Blood tests Fatigue and Physical function-HAQ-Di	Participants in the intervention group reported significantly greater improvements in pain, CRP levels, and function compared with the control group, which were clinically important.
Andonian et al [43] (USA) 16-wk RCT	n = 20 (mean age 66.7 y) Disease duration = 15.2 y	Two interventions CHAT intervention (n = 10) (1 nutrition and 1 exercise session) + 16 wk of regular check ins Vs SWET intervention (n = 10) -hypocaloric diet and aerobic and resistance exercise training		Disease activity- DAS28-CRP and DAS28-ESR. CRP and ESR-Commercial clinical analysis Pain, physical function, fatigue – patient-reported questionnaire	Participants assigned to the SWET intervention had significantly greater reductions in DAS28-CRP than those assigned to the CHAT intervention, with no significant differences in DAS28-ESR. Participants in the SWET intervention reported clinically important and statistically significant reductions in pain and CRP levels; however, results did not differ significantly compared with the CHAT intervention. The SWET intervention resulted in statistically significant and clinically important improvements in fatigue and function compared with the CHAT intervention.
Garcia-Morales et al [44] (Mexico City) 24-wk RCT	n = 130 (mean age 49 y) - Disease duration 8-17 y,	4 groups, Mediterranean diet +DEP (twice a week training sessions) (n = 32), DEP (n = 36), Mediterranean diet (n = 35) and control (n = 27)		Disease activity-DAS28 pain, function, and quality of life-SF-36 CRP and ESR- routinely automated analysers.	All participants had low disease activity at baseline. DAS28 did not differ between groups postintervention. Participants in the combined Mediterranean diet and DEP intervention reported statistically significant and clinically important improvements in pain and physical function; however, these improvements did not differ significantly between groups. The combined Mediterranean diet and DEP group and the DEP-only group had the greatest improvements in quality of life, which were statistically significant and clinically important.
Papandreou et al [46] (Greece) 12-wk single blind RCT	n = 40 female participants (mean age 34.03 ± 5.45) -RA diagnosis greater than 2 y	Intervention group (n = 20) receiving an isocaloric personalised Mediterranean Diet plan and physical activity advice vs a control group (n = 20) receiving usual care		Disease activity-DAS28 CRP- fasting blood samples.	Participants in the intervention group had significantly reduced disease activity after 3 mo compared to the control group. CRP levels did not differ between groups postintervention.
Torii et al [49] (Japan) 16-wk parallel RCT	n = 134 older adult participants (mean age 73.5) RA diagnosis 17.5 y	Intervention group (n = 69) Received an individualised Exercise programme including aerobic and resistance exercise and nutritional guidance versus a control group (n = 65) receiving nutritional guidance and usual care		Disease activity-DAS28-CRP Physical function and disability-HAQ Health-related quality of life-EQ-5D-3L and SF-36.	No statistically significant between-group differences in DAS28-CRP were observed. Disease activity remained stable in the intervention group throughout the study. HAQ scores did not differ significantly between groups after 16 wk. The intervention group demonstrated significant improvements in mental health–related quality of life (*P* < .05); however, no significant improvements were noted in overall quality of life.
Collins-Bennett et al [51] USA 2-y extension of the SWET RCT	n = 14 (n = 7 from CHAT intervention and n = 7 from SWET intervention) Mean age 68.9 y		Original SWET intervention consisted of hypocaloric diet guidance and aerobic and resistance exercises. During the follow-up period, participants in both the original SWET and CHAT intervention groups received SWET intervention materials	Disease activity- DAS28-CRP and DAS28-ESR CRP and ESR blood tests and clinical analysis Pain, fatigue, function- PROMIS bank questionnaires	Most participants (n = 10) reduced DAS28-CRP scores and had no increase in medication use. Participants who did not increase medication use (n = 12) observed significant improvements in DAS28-CRP and DAS28-ESR No significant changes in physical function, pain, or fatigue were observed between preintervention and extension study.

CHAT, Counselling Health As Treatment; CRP, C-reactive protein; DAS28, Disease Activity Score 28; DEP, Dynamic Exercise Programme; EQ-5D-3L, 3 generic health-related quality of life measures; ESR, erythrocyte sedimentation rate; HAQ-DI, Health Assessment Questionnaire Disability Index; n, number of participants; PROMIS, Patient Reported Outcome Measurement Information System; RA, rheumatoid arthritis; RCT, randomised controlled trial; SF-36, Short Form Survey; SWET, Supervised Weight loss and Exercise Training; VAS, visual analogue scale.

**Table 3:** Characteristics and main findings of studies assessing ≥2 lifestyle interventions

Study	Population	Intervention	Outcome measures	Key findings
Kurt et al [45] (Germany) 12-wk single blind RCT	n = 180 (mean age 53.3 ± 11.7 y, mean disease duration 8 y) living with inflammatory arthritis, including RA (n = 109), psoriatic arthritis (n = 32) and ankylosing spondylitis (n = 17).	Intervention group (n = 74) receiving lifestyle counselling, including adhering to a healthy Mediterranean Diet, partaking in sports and physical activity, and advice on mental health strategies and smoking cessation vs a control group (n = 84) receiving usual care.	Disease activity—Disease activity index (RADAI) and Disease Activity Score (DAS). Quality of life and function—SF-36.	Participants in the intervention group observed reductions in disease activity compared with the control group; however, results were not statistically significant or clinically important. No individual data were reported for people living with RA on secondary outcome measures. Overall, no significant changes were noted for health-related quality of life or physical function (*P* > .05).
Walrabenstein et al [47] (The Netherlands) 16-wk parallel blind RCT	n = 77 (mean age 55 y) -Disease duration 9 y.	Intervention group (n = 40) receiving education on plant-based eating and physical activity, psychoeducation on stress management, and coaching on sleep vs control group (n = 37) receiving usual care.	Disease activity-DAS28-ESR physical function, fatigue, and pain—Dutch-Flemish Patient Reported Outcomes Measurement Information System (PROMIS)	Participants in the intervention group had a significantly greater decrease in disease activity compared to the control group (*P* < .0001) Pain, fatigue, function, and CRP levels improved; however, changes were neither statistically significant nor clinically important.
Yousefi et al [48] (Iran) 10-wk RCT Baştürk et al [50] (Turkey) A 4-wk, 8-session RCT with a 6-mo follow-up.	n = 57 (mean age = 50.2 y) -RA diagnosis greater than 6 mo (n = 33) (mean age = 49.9 y) -mean disease duration: ≈ 7.3 y	- Group 1 (n = 19) – cognitive behavioural session that includes stress management, the benefits of sufficient sleep, and ways to improve sleep - Group 2 (n = 19) mindfulness-based stress reduction, including meditation, mindfulness, suggestions for exercise, group 3 (n = 19) control group receiving standard drug treatment. Lifestyle intervention group (n = 17) receiving a lifestyle intervention incorporating stress management, sleep hygiene, physical activity, and social participation versus control group (n = 16) receiving usual pharmacological care.	Disease activity-DAS28 Fatigue-Chalder fatigue scale-CFS activity-DAS28 Pain-VAS Fatigue-BRAF-MDQ Health-related quality of life-RAQoL	Both the MBSR and CBT intervention groups achieved reductions in disease activity, moving from moderate to low disease activity classifications; however, neither intervention demonstrated a statistically significant effect on disease activity. Both the MBSR and CBT groups demonstrated statistically significant reductions in fatigue compared with the control group; however, changes were not clinically important. Participants in the intervention group demonstrated significant reductions in disease activity from baseline to 6-mo follow-up and had significantly lower disease activity compared with the control group at 6-mo follow-up. However, the change was not clinically important. Participants in the intervention group demonstrated significantly greater improvements in pain, fatigue, and quality of life compared with the control group at postintervention and 6-mo follow-up. The change in fatigue was clinically important.
Wagenaar et al [52] (The Netherlands) 12-mo extension of the PFJ RCT Wagenaar et al [53] (The Netherlands) 24-mo extension of the PFJ RCT	n = 65 from the 77 participants who completed the PFJ trial. (n = 48) from the 77 participants who completed the original PFJ trial.	Interventions included stress management, diet, and physical activity interventions following on from the RCT study. After the Plants for Joints RCT, the control group received the intervention. Following completion of the PFJ trial, control group participants also received the intervention. All participants were followed as a single intervention cohort for 2 y, supported by biannual visits and adherence-promoting webinars	Disease activity-DAS28-ESR physical function, pain, fatigue—Dutch-Flemish PROMIS	The original control group showed improvements in DAS28 after receiving the intervention during the one-year follow-up, and when combined with the original intervention group, DAS28 decreased significantly (*P* < .001). Significant reductions in CRP levels were highlighted (*P* = .03). No significant changes in physical function, fatigue or pain were noted at the end of the extension study. Improvements in disease activity were maintained, with significantly lower disease activity compared with baseline. CRP levels also remained significantly lower than baseline.

BRAF-MDQ, Bristol Rheumatoid Arthritis Fatigue Multi-Dimensional Questionnaire; CBT, cognitive behavioural therapy; CFS, Chalder Fatigue Scale; CRP, C-reactive protein; DAS, Disease Activity Score; DAS28-ESR, Disease Activity Score in 28 joints-erythrocyte sedimentation rate; ESR, erythrocyte sedimentation rate; MBSR, mindfulness-based stress reduction; n, number of participants; PFJ, Plants for Joints; PROMIS, Patient-Reported Outcomes Measurement Information System; RA, rheumatoid arthritis; RADAI, Rheumatoid Arthritis Disease Activity Index; RAQoL, Rheumatoid Arthritis Quality of Life; RCT, randomised controlled trial; SF-36, 36-Item Short Form Health Survey; VAS, visual analogue scale.

**Table 4:** Quantitative outcomes across included studies (mean [SD] or median [min-max] at baseline and endpoint)

Study	Time point	Disease activity	Inflammatory marker	Pain	Fatigue	Function
Abdel-Aal et al [42]	Baseline^a^		CRP: I: 8.0 (1.64) C: 8.37 (1.94)	I: 72.33 (9.71), C:72.67 (8.68)		I: 2.17 (0.49), C: 2.28 (0.41)
	Postintervention		I: 4.75 (0.83)^c^ C: 8.0 (1.7)	I: 29.33 (10.48)^c^ C: 43.33 (9.59)^c^		I: 0.94 (0.36)^c^ C: 1.92 (0.64)^b^
Andonian et al [43]	Baseline	DAS28-ESR: CHAT(C): 3.6 (1.0), SWET (I): 3.3 (1.2) DAS28-CRP: C: 3.1 (1.0), I: 2.9 (1.2)	CRP: C: 8.2 (13.0), I: 4.7(9.5), ESR: C: 26.0 (24.0) I: 22.3 (18.0)	C: 46.5 (6.1) I: 46.3 (5.8)	C: 49.0 (9.1) I: 49.0 (6.3)	C: 41.7 (5.0) I: 42.3 (6.3)
	Postintervention	DAS28-ESR: C: 3.3 (0.9) I: 2.8 (1.0) DAS28-CRP: C: 2.9(0.8) I: 2.1(0.9)^b^	CRP: C: 9.3(11.5) I: 3.6 (4.4)^b^ ESR: C: 29.5 (31.1) I: 26.5 (17.6)	C: 45.7 (4.0) I: 41.7 (7.9)^b^	C: 52.0 (6.0) I: 44.0 (9.9)	C: 41.7 (6.4) I: 48.1 (6.5)^b^
Garcia-Morales et al [44]	Baseline	MD+DEP (I): 2.47(0.79) DEP (C1): 2.67 (0.90) MD (C2): 2.2(1.06) C:2.59(0.88)		I: 41 (22.0-61.7) C1: 51 (32-62) C2: 62 (51-81) C: 51.5 (30.7-66.5)		I: 50 (31.2-68.7) C1: 65 (50-80) C2: 85 (55-95) C: 70 (40-81.2)
	Postintervention	Postintervention disease activity values not reported		I: 61.5 (44.3-83)^b^ C1: 52 (51-62) C2: 62 (51-84) C: 52 (31.5-72)		I: 77.5 (51.3-88.8)^b^ C1: 75 (45-90)^b^ C2: 82.5 (60-91.3) C: 70 (45-90)
Papandreou et al [46]	Baseline	I: 2.83 (0.19) C: 2.81 (0.2)	CRP: I: 0.71 (0.11), C: 0.77 (0.12),			
	Postintervention	I: 2.71 (0.14)^c^ C: 2.80 (0.17)	I: 0.72 (0.11), C: 0.75 (0.11)			
Walrabenstein et al [47]	Baseline	I: 3.90 (0.7), C: 3.78 (0.7),	CRP: I: 4.3 (4.1), C: 3.4 (3.9) ESR: I: 17(18), C: 20 (15)	I: 58 (7) C: 60 (6)	I: 53 (12) C: 58 (6)	I: 44 (5), C: 43(5)
	Postintervention	I: 2.88 (1.1)^c^ C: 3.79 (1.0)	CRP: I: 3.8 (4.4), C: 4.8 (8.3) ESR: I: 17(14) C: 24 (16)	I: 55 (7) C: 56 (6)	I: 52(7) C: 55(8)	I: 46(8) C: 44(5)
Yousefi et al [48]	Baseline	MBSR (I): 3.58, (0.77) CBT (C): 3.50(0.87) C2: 3.64 (1.12)			I: 16 (6.14), C: 16.74 (7.95), C2: 17.68 (6.63)	
	Postintervention	I:2.69 (0.75) C: 2.81 (0.96) C2: 3.07 (1.01)			I: 11.68 (5.01) C: 8.58(5.54) C2: 17.95 (6.35)	
Torii et al [49]	Baseline	I: 2.1 (0.8) C: 2.1 (0.7)				I: 42.5 (11.6), C:42.8 (11.0)
	Postintervention	I: 3.2 (0.8) C: 3.0 (0.9)				I: 43.2 (12.7) C: 42.8 (11.7)
Baştürk et al [50]	Baseline:	I: 3.98 (0.69) C: 4.20 (0.66)		I: 5.71 (1.49), C: 5.79 (1.05)	I: 32.53 (17.39), C: 29.0 (14.87),	
	Postintervention	I: 3.41 (0.78)^c^ C: 4.29 (0.71)		I: 1.82 (0.81)^c^ C: 5.79 (0.80)	I: 5.18 (5.35)^c^ C: 26.94 (15.13)	
Kurt et al [45]	Baseline:	I: 2.52 (0.4-7.5) C: 2.55 (0.96-19.2)				
	Postintervention	No postintervention data reported.				
Collins-Bennett et al [51]	Baseline	DAS28-ESR: 3.7 (1.0) DAS28-CRP: 3.2 (1.1)	CRP: 8.9 (12.1) ESR: 27.9 (24.3)	46.1 (6.3)	49.1 (7.2)	42.2 (5.7)
	Extension study	DAS28-ESR: 2.4 (1.2)^c^ DAS28-CRP: 2.2 (0.9)^c^	CRP: 4.8 (5.6)^c^ ESR: 18.8 (27.9)	45.4 (9.7)	47.7 (9.1)	44.0 (8.5)

C, control; CBT, cognitive behavioural therapy; CRP, C-reactive protein; DAS28, Disease Activity Score in 28 joints; DAS28-CRP, Disease Activity Score in 28 joints-C-reactive protein; DAS28-ESR, Disease Activity Score in 28 joints-erythrocyte sedimentation rate; DEP, dynamic exercise programme; ESR, erythrocyte sedimentation rate; I, intervention group; MBSR, mindfulness-based stress reduction; MD, Mediterranean diet.

a Baseline measurements represent preintervention values.

b Within-group change was statistically significant (*P* < .05).

c Within-group change was statistically significant (*P* < .001).

### Primary outcome: disease activity

In the MADEIRA RCT, participants assigned to the Mediterranean diet and physical activity intervention group (n = 20) had a significant reduction in disease activity after 12 weeks compared with the control group (DAS28 change: −0.12 vs −0.01; between-group difference: not reported; *P* < .001) [46]. The intervention within-group change was below the MCID of 1.2 points and therefore did not represent a clinically important improvement [54]. In the PFJ RCT (n = 77), the intervention group demonstrated a significantly greater reduction in disease activity than the control group (DAS28 change: −1.02 vs 0.01; between-group mean difference: 0.90, 95% CI: 0.41-1.29; *P* < .001), with further reductions at 1-year follow-up [47,52]. However, the within-group intervention change was not clinically important [54]. The 2-year follow-up study also reported significantly lower disease activity across all participants compared with baseline values (95% CI: −1.2 to −0.6; *P* < .0001) [53]. In an RCT, participants receiving a combined diet, exercise, mental health, and smoking cessation intervention reported a greater reduction in disease activity than those in the control group (DAS28 change: −0.6 vs −0.4; between-group difference: not reported; *P* = .055). However, the within-group change in the intervention group was neither statistically significant nor clinically important [45,54]. In addition, a 16-week study comparing a single exercise and nutrition session (Counselling Health As Treatment [CHAT]) with a hypocaloric diet and aerobic training intervention (SWET, n = 10) found a greater reduction in DAS28-CRP in the SWET group (−0.8 vs −0.2; between-group difference: not reported; *P* = .04) [43]. However, the within-group SWET intervention change was not clinically important [54]. In the 2-year follow-up, participants from the SWET and CHAT groups who did not increase RA medication use (n = 12) demonstrated significant within-group reductions in DAS28-CRP (−1.0 ± 0.9; *P* = .002) and DAS28-ESR (−1.3 ± 0.9; *P* = .0004) compared with preintervention [51]. A recent RCT reported a significantly greater reduction in disease activity from baseline to 6-month follow-up among participants receiving an intervention (n = 17) combining stress management, sleep hygiene, physical activity, and social participation compared with usual care (DAS28 change: −0.57 vs +0.09; between-group difference: not reported; *P* = .002); however, the within-group intervention change was not clinically important [50,54]. Across these 5 studies, baseline and postintervention disease activity are presented in Table 4, indicating that some studies had low baseline disease activity, which may have limited the potential for further improvement.

Conversely, a 6-month RCT comparing a Mediterranean diet intervention (n = 26), a dynamic exercise programme (DEP) (n = 30), or both (n = 28) showed no postintervention differences in disease activity; however, all groups reported low DAS at baseline [44]. An RCT comparing mindfulness-based stress reduction (MBSR, n = 19) with cognitive behavioural therapy (CBT, n = 19) observed reductions in disease activity, but results were neither statistically significant nor clinically important [48]. Moreover, a recent RCT showed no statistically significant differences in DAS28-CRP after 16 weeks between the intervention group (n = 69) receiving an aerobic and resistance exercise programme with nutritional guidance compared with the control group; however, participants had low disease activity at baseline [49].

### Secondary outcome measures

#### Inflammatory markers

A significant decrease in CRP levels was noted in individuals assigned to a 12-month Mediterranean diet and exercise intervention (n = 30) compared with the control group (CRP change: −3.25 vs −0.37 mg/L; between-group difference: not reported; *P* < .001), and the within-group change in the intervention group was clinically important (MCID for CRP: 1.02 mg/L) [42,55]. In addition, in the SWET RCT, participants assigned to the SWET intervention experienced a significant and clinically important within-group reduction in CRP levels (−1.1 mg/L, *P* = .04), whereas no significant change was observed in the CHAT group (+1.1 mg/L, *P* = .91) [42,43,55]. However, the change did not differ significantly between groups (*P* = .24) [43]. The MADEIRA RCT found no significant differences in CRP after 12 weeks [46]. The PFJ RCT reported nonsignificant reductions in CRP and ESR levels in the intervention group, neither of which was clinically important [47]. The 1-year follow-up study reported a modest reduction in CRP (−0.5 mg/L) [52]. At the 2-year follow-up, CRP levels were significantly lower than baseline values [53].

#### Pain and fatigue

One RCT found that individuals receiving a combined Mediterranean diet and exercise intervention reported greater significant reductions in pain than those receiving an exercise intervention alone (VAS change: −43.0 vs −29.34; between-group mean difference: −14.0; *P* < .001), with the within-group intervention change being clinically important (MCID for VAS pain: 10-20 mm) [39,42]. Another RCT found that a combined Mediterranean diet and DEP intervention had the greatest improvement in pain compared with the DEP, Mediterranean diet, and control groups (36-Item Short Form Survey [SF-36] change: +20 vs +10 vs +6.5 vs 0, respectively) [44]. The within-group change in the combined intervention group was statistically significant and clinically important (MCID for SF-36 bodily pain: 4.9 points); however, the between-group difference was not statistically significant (*P* = .07) [39,44].

The PFJ RCT observed slight reductions in fatigue and pain levels, but results were not statistically significant [47]. However, the 1-year follow-up study indicated lower pain interference scores than at baseline (−3, *P* = .001), whereas fatigue levels remained unchanged [52]. A combined stress management, sleep hygiene, physical activity, and social participation intervention demonstrated significantly greater reductions in pain (VAS change: −3.89 vs 0; between-group difference: not reported) and fatigue (Bristol Rheumatoid Arthritis Fatigue Multi-Dimensional Questionnaire [BRAF-MDQ] change: −27.35 vs −2.06; between-group difference: not reported) than the control group at the 6-month follow-up (between-group *P* = .002), with the within-group intervention change in fatigue being clinically important (MCID: 17.5% improvement in BRAF-MDQ) [50,56]. The SWET intervention resulted in a clinically important and significant within-group reduction in pain from baseline to postintervention (Patient Reported Outcome Measurement Information System [PROMIS]: −4.6, *P* = .04; approximate MCID: 2 points); however, the change did not differ significantly from CHAT (between-group difference: not reported; *P* = .11) [43,57]. However, the SWET intervention resulted in a significantly greater reduction in fatigue than CHAT, which was clinically important (PROMIS: −5.0 vs +3.0; between-group difference: not reported; *P* = .02; MCID: 2 points) [43,57]. Participants in the 2-year extension study showed no significant changes in pain or fatigue from preintervention to follow-up [51]. An RCT comparing MBSR and CBT interventions found that both interventions resulted in significant reductions in fatigue compared with control (*P* < .001), with CBT (n = 19) yielding a greater reduction than MBSR (Chalder Fatigue Scale (CFS) change: −8.16 vs −4.32; between-group differences: not reported), while fatigue levels slightly increased in the control group (+0.27) [48]. However, within-group intervention changes were not clinically important (MCID CFS: 9.9 points) [39].

#### Physical function

The SWET intervention resulted in significant and clinically important improvements in physical function compared with the CHAT intervention (PROMIS Physical Function change: +5.8 vs 0; between-group difference: not reported; *P* = .01; MCID: 2 points) [39,43]; however, improvements were not maintained at 2-year follow-up [51]. The PFJ RCT observed no significant effects on physical function or notable changes in the 1-year extension study [47,52]. A combined aerobic/resistance exercise and nutritional guidance intervention showed no significant improvements in physical function after 16 weeks compared with the control group [49]. However, a combined Mediterranean diet and aerobic/muscle strengthening exercise intervention yielded significantly greater improvements in function compared with the control group (HAQ-DI change: −1.23 vs −0.37; between-group mean difference: −0.98, 95% CI −1.24 to −0.71; *P* < .001); and the within-group intervention change was clinically important (Health Assessment Questionnaire Disability Index MCID: −0.22) [39,42]. Similarly, participants assigned to a combined Mediterranean diet and DEP intervention (n = 32) exhibited the greatest clinically important improvement in function (SF-36 change: +20 vs +10 vs +2.5 vs 0, respectively; MCID: 7.1), with a significant between-group difference observed compared with control (*P* = .005) [39,44].

#### Quality of life

One RCT reported that the combined Mediterranean diet and DEP and DEP interventions demonstrated the greatest within-group clinically important improvements in quality of life compared with the Mediterranean diet and control groups (SF-36 change: +15 vs +15 vs +3.5 vs −4.6, respectively; between-group difference: not reported; *P* = .01, MCID: 5 points) [39,44]. Participants in the combined sleep hygiene, stress management, physical activity, and social participation intervention demonstrated significantly greater improvements in quality of life compared with the control group (Rheumatoid Arthritis Quality of Life [RAQoL] change: −1.24 vs +1.50; between-group difference: not reported; *P* < .001); however, the within-group intervention change was not clinically significant (MCID RAQoL: 1.7) [39,50]. In contrast, a combined exercise and nutrition intervention did not significantly improve overall health-related quality of life but significantly improved mental health–related components, including vitality (+3.5, 95% CI: 0.8-6.1) and mental health (+2.8, 95% CI: 0.2-5.5) [49].

#### Results of risk of bias assessment

Of the 9 RCTs assessed using RoB-2, 3 were judged to have a low risk of bias [42,44,47], 5 had some concerns [43,45,46,49,50], and 1 had a high risk [48] due to concerns with the randomisation process and deviations from intended interventions, limiting confidence in findings*.* However, this study was included in the analysis because this review was descriptive in nature. Figure 2 presents the risk of bias assessments for the RCTs.

**Figure 2:**
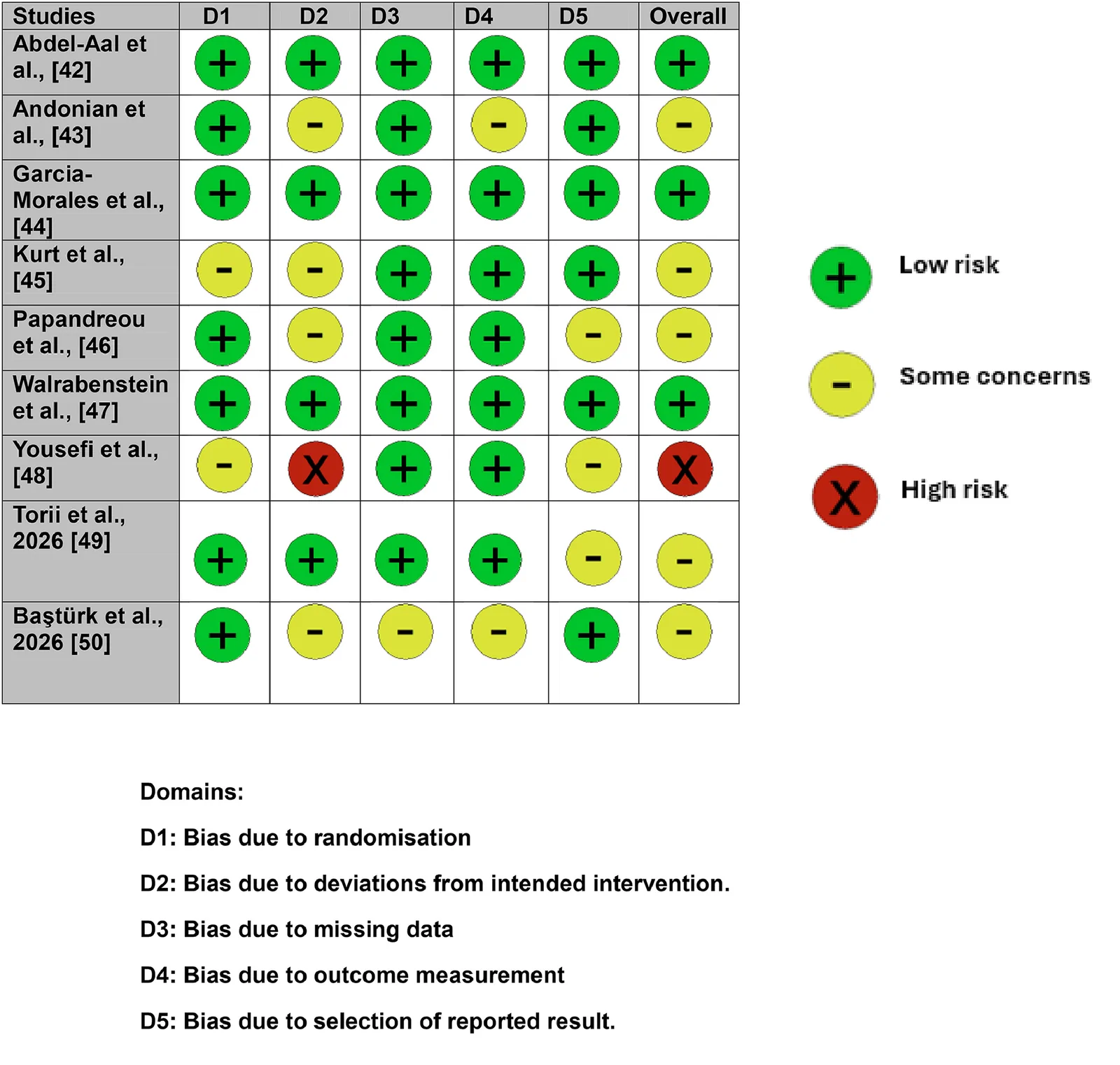
Quality assessment of randomised controlled trials. Domains: D1: bias due to randomisation. D2: bias due to deviations from the intended intervention. D3: bias due to missing data. D4: bias due to outcome measurement. D5: bias due to the selection of reported result.

#### Results of GRADE assessment

The GRADE approach indicated low and very low certainty of evidence for multifactorial lifestyle interventions across disease activity, pain, physical function, fatigue, inflammatory markers, and quality of life outcome measures (Table 5). Although many studies demonstrated improvements, the risk of bias due to methodological limitations, imprecision from small sample sizes, and inconsistency in results reduced confidence in the overall findings.

**Table 5:** GRADE certainty ratings for each outcome

Outcome	No. of studies	Effect of intervention	Certainty of evidence (GRADE)	Reasons for downgrading
Disease activity	11 studies	Several studies reported statistically significant reductions in disease activity following multifactorial lifestyle interventions, with further reductions reported at follow-up; however, changes were not clinically important. Three studies reported no statistically significant reductions, although baseline disease activity was low	⨁⨁◯◯ Low	Downgraded for risk of bias (several studies with some concerns/high risk) and imprecision (small sample sizes and wide uncertainty)
Pain	7 studies	Multifactorial lifestyle interventions generally reduced pain compared with controls or single interventions, with clinically important improvements noted in some studies. However, some findings were inconsistent across studies, with some reporting no statistically significant effects and follow-up studies reporting no further improvements	⨁⨁◯◯ Low	Downgraded for risk of bias and imprecision due to small trials and inconsistent estimates
Physical function	8 studies	Several studies reported improvements in physical function, particularly when diet and exercise were combined, with some noting clinically important improvements. However, other studies found no statistically significant changes, and follow-up studies reported no further improvements, resulting in variability across findings	⨁⨁◯◯ Low	Downgraded for risk of bias and imprecision
Fatigue	6 studies	Some interventions demonstrated statistically significant and clinically important reductions in fatigue, including exercise- and behaviour-based interventions. However, evidence was inconsistent across studies, with some follow-up studies reporting no further improvements	⨁⨁◯◯ Low	Downgraded for risk of bias and imprecision
Inflammatory markers	6 studies	Results were mixed. Some studies reported statistically significant reductions in CRP, with some findings being clinically important; however, other studies reported no statistically significant or clinically important reductions	⨁◯◯◯ VERY LOW	Downgraded for risk of bias, inconsistency between studies, and imprecision
Quality of life	3 studies	Evidence suggested possible improvements in health-related quality of life and mental health; however, findings were inconsistent and based on a small number of studies	⨁◯◯◯ VERY LOW	Downgraded for risk of bias, inconsistency, and imprecision

CRP, C-reactive protein; GRADE, Grading of Recommendations Assessment, Development, and Evaluation.

## DISCUSSION

This review aimed to examine the evidence of the effects of multifactorial lifestyle interventions on core outcomes, including disease activity, inflammatory markers, fatigue, function, pain, and quality of life in adults with RA. The findings of this review provide some evidence that multifactorial lifestyle interventions may influence disease activity and other core outcomes in RA; however, results varied across studies and outcome measures.

Five studies reported significant reductions in disease activity after lifestyle interventions, whereas 3 studies did not report statistically significant changes. Statistically significant improvements in disease activity did not translate into clinically important changes. The variability in findings may be partly explained by lower baseline disease activity in studies reporting nonsignificant and non-clinically relevant findings, as well as smaller sample sizes. These factors may have limited the ability of studies to detect further improvements from already low levels of disease activity and reduced their statistical power to detect significant changes. Furthermore, 1 study reported a shift from moderate to low disease activity after the intervention, despite the change not reaching statistical significance. This highlights potential variability in disease activity outcomes across studies and indicates that nonsignificant findings may not necessarily indicate an absence of improvement. Findings for secondary outcomes were similarly variable, with some studies reporting significant improvements in inflammatory markers, pain, fatigue, function, and quality of life, whereas others did not. However, many studies reported clinically and statistically significant improvements, particularly for pain and fatigue.

A variety of lifestyle factors were assessed across studies, with diet and exercise emerging as the predominantly researched lifestyle factors, whereas smoking cessation, stress management, and sleep were examined in fewer trials. The higher prevalence of diet and exercise interventions reflects the broader evidence base supporting the role of diet and exercise in improving RA outcomes [14,23,58]. These lifestyle factors are also represented in the 2023 European Alliance of Associations for Rheumatology recommendations on lifestyle behaviours in rheumatic and musculoskeletal diseases, alongside smoking cessation and minimising alcohol consumption, to support overall health and disease outcomes [59].

Evidence is also growing regarding the role of specific dietary components and supplements, with 2 trials investigating exercise interventions alongside curcumin supplementation [60] and green tea [61], respectively, reporting significant improvements in disease activity, inflammatory markers, physical function, and pain. Although not eligible for inclusion in this review, these studies provide further evidence of the potential benefits of combined lifestyle approaches while highlighting the predominance of diet and exercise interventions within the existing evidence base [60,61].

Consistent with the growing evidence examining diet and exercise in RA, 1 previous systematic review and meta-analysis of individuals with inflammatory arthritis, including RA, reported that combined dietary and exercise interventions significantly improved physical function; however, the review also reported significant increases in ESR after the interventions [34]. In the present review, which included a broader range of lifestyle factors, no overall increases in inflammatory markers were observed. However, direct comparisons regarding pain, disease activity, and fatigue cannot be made because these outcomes were not evaluated in the previous review. Given the variability observed across outcome measures, future studies should further investigate the effects of multifactorial lifestyle interventions on core outcomes recommended by OMERACT, including pain, fatigue, and disease activity, to facilitate more robust comparisons and potentially inform future guideline development [38].

Multifactorial lifestyle interventions have shown effectiveness in other chronic conditions, including chronic obstructive pulmonary disease and asthma, including a systematic review highlighting that multifactorial lifestyle interventions, including diet, exercise, and smoking cessation, can improve respiratory symptoms, quality of life, body mass index, and exercise capacity [30]. Furthermore, previous reviews on metabolic syndrome and obesity highlighted that multicomponent interventions were more effective at reducing risk factors than individual lifestyle interventions [62,63]. This was demonstrated in 2 studies included in the present review, in which combined Mediterranean diet and exercise interventions resulted in greater improvements in outcome measures than either intervention alone [42,44].

There are several potential mechanisms through which multifactorial lifestyle interventions may result in greater benefits, potentially through exerting effects across similar inflammatory pathways and processes. For example, proinflammatory dietary patterns, poor sleep, smoking, and excessive alcohol consumption may contribute to proinflammatory signalling and increased levels of proinflammatory cytokines, including TNF-α and interleukin-6 [21,22,64,65]. Conversely, physical activity and dietary patterns rich in anti-inflammatory components may attenuate proinflammatory pathways, whereas physical activity can also stimulate the release of myokines that may contribute to anti-inflammatory effects [19,20,64]. Another potential mechanism may involve the ability of physical activity and polyphenols found in anti-inflammatory dietary patterns to modulate the nuclear factor kappa B signalling pathway, which regulates inflammatory gene expression [20,66].

A further potential mechanism relates to oxidative stress, as lifestyle factors such as smoking and physical inactivity may contribute to increased reactive oxygen species, which can interact with inflammatory signalling pathways [20,67]. Taken together, these overlapping mechanisms of action provide plausible explanations for why combined lifestyle interventions may result in greater effects on the underlying inflammatory pathophysiology of RA and, consequently, greater improvements in disease severity and management. In addition, physiological interconnections among lifestyle factors, including sleep, stress, exercise, and nutrition, demonstrate how impairments in one domain can adversely affect others, reinforcing the rationale for multifactorial lifestyle interventions [[68], [69], [70]].

However, as the present review aimed to examine the effects of multifactorial lifestyle interventions on core outcomes rather than their underlying biological mechanisms, and the observed effects are based on 2 studies, these potential mechanisms remain theoretical [42,44]. Further research is warranted to investigate the biological mechanisms underlying multifactorial lifestyle interventions and determine whether any greater effects arise through synergistic or additive effects.

It is also crucial to consider the feasibility and practicality of multifactorial lifestyle interventions, given their labour-intensive nature, costs, and inherent challenges of sustaining lifestyle changes [45,[71], [72], [73]]. A cost-effective analysis of the PFJ trial demonstrated that the intervention had a high probability of being cost-effective from a societal perspective, suggesting that such interventions may offer broader societal benefits and potentially reduce disease burden; however, further research is required to determine their cost-effectiveness within healthcare systems [74]. Furthermore, a 16-week feasibility study incorporating exercise, nutrition, and behaviour counselling found the intervention to be acceptable and feasible for adults with RA, and resulted in significant improvements in diet composition, physical function, and sitting time [75]. Together, these studies contribute to the evidence base surrounding the broader implementation of multifactorial lifestyle interventions, including feasibility, acceptability, and cost-effectiveness, highlighting considerations beyond clinical outcomes alone.

Despite some studies reporting statistically significant and clinically important changes in outcome measures after the interventions, the overall findings require careful consideration and interpretation, given the low to very low certainty of evidence, attributed to risk of bias, imprecision, and inconsistency. Most studies received moderate or high risk of bias judgements primarily related to deviations from intended interventions and selection of the reported results. These concerns may reduce confidence that the reported effects can be directly attributed to the lifestyle interventions under investigation [40]. Imprecision related to small sample sizes may further limit the generalisability of findings to broader and more diverse populations of individuals living with RA and different clinical settings [76]. Furthermore, variability in results may make it more difficult to translate these findings into clinical recommendations and effectively implement multifactorial lifestyle interventions into routine RA care [40]. These limitations highlight the need for more robustly designed studies to establish their effectiveness.

Lifestyle medicine approaches are increasingly recognised in chronic disease management, with growing integration into healthcare delivery, medical curricula, and the establishment of lifestyle medicine organisations [15,77,78]. However, this review identified a shortage of high-quality multifactorial lifestyle interventions in RA, with limited focus on factors including sleep, social connection, stress, and alcohol consumption, despite evidence demonstrating their individual influence on RA outcomes. Given the potential for lifestyle medicine interventions to reduce disease burden and improve quality of life, further high-quality research is needed to evaluate integrated approaches to RA care that address underrepresented lifestyle factors and broader determinants of health.

This review has limitations. Restricting the population to adults with RA led to the exclusion of studies involving broader inflammatory arthritis populations without RA-specific data, reducing the number of eligible studies. The certainty of evidence was low, with several studies characterised by small sample sizes and nonsignificant findings. Substantial heterogeneity in intervention characteristics and outcome measures limited the ability to conduct a meta-analysis and prevented definitive conclusions regarding the most effective lifestyle interventions. Several studies also exhibited methodological limitations, including unmeasured adherence, high attrition, and reliance on subjective outcome measures, further reducing confidence in the findings. Furthermore, because of variability in interventions, it is unclear which aspects of lifestyle interventions, or which combination of lifestyle interventions, contribute to the observed findings.

Nonetheless, this review has notable strengths. It employed rigorous methodology, including predefined eligibility criteria, a comprehensive literature search, and quality assessment using established tools. It also addresses a wide range of OMERACT core outcomes, including physical function, pain, fatigue, acute phase reactants, and health-related quality of life, facilitating future comparisons. To the authors’ knowledge, this is the first review to synthesise the evidence on multifactorial lifestyle interventions across RA outcomes, providing a foundation for future research.

## CONCLUSION

This review synthesised evidence from clinical trials examining the effects of multifactorial lifestyle interventions on RA outcomes. Multifactorial lifestyle interventions may improve disease activity and other core outcome measures in RA; however, findings were inconsistent across studies. Variations in baseline characteristics, small sample sizes, methodological limitations, and low certainty of evidence limit the interpretation of the findings and highlight the need for high-quality research to evaluate the effectiveness, feasibility, acceptability, and implementation of multifactorial lifestyle interventions in routine RA care.

## Appendix Supplementary materials

Image, application 1

## Footnotes

Supplementary material associated with this article can be found in the online version at doi:10.1016/j.ero.2026.100254.
