# Supplementary material for: Multifactorial lifestyle interventions in rheumatoid arthritis: a systematic literature review
Source: EULAR Rheumatol Open. 2026 Oct 8;2(4):100254. doi: 10.1016/j.ero.2026.100254 (PMC13676706; doi:10.1016/j.ero.2026.100254)
Supplement: Supplementary file 1 [file mmc1.docx]

**Systematic Review Search Strategy across 5 databases**

**1. PubMed**

1. "Rheumatoid Arthritis"[MeSH]

2. "rheumatoid arthritis"[tiab]

3. RA[tiab]

4. diet[tiab]

5. nutri*[tiab]

6. "physical activ*"[tiab]

7. exercis*[tiab]

8. sleep[tiab]

9. stress[tiab]

10. resilience[tiab]

11. smok*[tiab]

12. "substance use"[tiab]

13. alcohol[tiab]

14. lifestyle[tiab]

15. "combined lifestyle"[tiab]

16. "multicomponent lifestyle"[tiab]

17. "multiple lifestyle"[tiab]

18. or/1-3 [RA terms]

19. or/4-17 [Lifestyle terms]

20. 18 and 19

21. Humans[Mesh]

22. English[lang]

23. ("2014/01/01"[PDAT] : "2025/12/31"[PDAT])

24. Randomized Controlled Trial[pt] OR Observational Study[pt]

25. 20 AND 21 AND 22 AND 23 AND 24

CINAHL/EBSCOhost:

1. MH "Rheumatoid Arthritis"

2. TI "rheumatoid arthritis" OR AB "rheumatoid arthritis"

3. TI RA OR AB RA

4. MH Diet

5. MH Nutrition

6. MH "Physical Activity"

7. MH Exercise

8. MH Sleep

9. MH Stress

10. MH Smoking

11. MH "Substance Use"

12. MH Alcohol

13. MH "Life Style"

14. TI diet OR AB diet

15. TI nutri* OR AB nutri*

16. TI "physical activ*" OR AB "physical activ*"

17. TI exercis* OR AB exercis*

18. TI sleep OR AB sleep

19. TI stress OR AB stress

20. TI resilience OR AB resilience

21. TI smok* OR AB smok*

22. TI "substance use" OR AB "substance use"

23. TI alcohol OR AB alcohol

24. TI lifestyle OR AB lifestyle

25. TI "combined lifestyle" OR AB "combined lifestyle"

26. TI "multicomponent lifestyle" OR AB "multicomponent lifestyle"

27. TI "multiple lifestyle" OR AB "multiple lifestyle"

28. or/1-3 [RA terms]

29. or/4-27 [Lifestyle terms]

30. 28 and 29

31. Limit 30 to Humans

32. Limit 30 to English

33. Limit 30 to Publication Years 2014–2025

34. Limit 30 to RCTs OR Observational Studies

Embase:

1. 'rheumatoid arthritis'/exp

2. 'rheumatoid arthritis':ti,ab

3. RA:ti,ab

4. 'diet'/exp

5. 'nutrition'/exp

6. 'exercise'/exp

7. 'motor activity'/exp

8. 'sleep'/exp

9. 'stress'/exp

10. 'smoking'/exp

11. 'substance use'/exp

12. 'alcohol'/exp

13. 'lifestyle'/exp

14. diet:ti,ab

15. nutri*:ti,ab

16. "physical activ*":ti,ab

17. exercis*:ti,ab

18. sleep:ti,ab

19. stress:ti,ab

20. resilience:ti,ab

21. smok*:ti,ab

22. "substance use":ti,ab

23. alcohol:ti,ab

24. lifestyle:ti,ab

25. "combined lifestyle":ti,ab

26. "multicomponent lifestyle":ti,ab

27. "multiple lifestyle":ti,ab

28. or/1-3 [RA terms]

29. or/4-27 [Lifestyle terms]

30. 28 and 29

31. Limit 30 to Humans

32. Limit 30 to English

33. Limit 30 to Publication Years 2014–2025

34. Limit 30 to RCT OR Observational Studies

SPORTDiscus:

1. TI "rheumatoid arthritis" OR AB "rheumatoid arthritis"

2. TI RA OR AB RA

3. TI diet OR AB diet

4. TI nutri* OR AB nutri*

5. TI "physical activ*" OR AB "physical activ*"

6. TI exercis* OR AB exercis*

7. TI sleep OR AB sleep

8. TI stress OR AB stress

9. TI resilience OR AB resilience

10. TI smok* OR AB smok*

11. TI "substance use" OR AB "substance use"

12. TI alcohol OR AB alcohol

13. TI lifestyle OR AB lifestyle

14. TI "combined lifestyle" OR AB "combined lifestyle"

15. TI "multicomponent lifestyle" OR AB "multicomponent lifestyle"

16. TI "multiple lifestyle" OR AB "multiple lifestyle"

17. or/1-2 [RA terms]

18. or/3-16 [Lifestyle terms]

19. 17 and 18

20. Limit 19 to Humans

21. Limit 19 to English

22. Limit 19 to Publication Years 2014–2025

23. Limit 19 to RCTs OR Observational Studies

| **Database** | **Search Date** | **Search String** |
| --- | --- | --- |
| PubMed | Jan–Feb 2024; updated Dec 2025 | Aug 2025 ("rheumatoid arthritis"[MeSH] OR "rheumatoid arthritis"[tiab]) AND (diet[tiab] OR nutri*[tiab] OR "physical activ*"[tiab] OR exercis*[tiab] OR sleep[tiab] OR stress[tiab] OR resilience[tiab] OR smok*[tiab] OR "substance use"[tiab] OR alcohol[tiab] OR lifestyle[tiab] OR "combined lifestyle"[tiab] OR "multicomponent lifestyle"[tiab] OR "multiple lifestyle"[tiab]) |
| Embase | Jan–Feb 2024; updated Dec 2025 | ('rheumatoid arthritis'/exp OR 'rheumatoid arthritis':ti,ab) AND (diet:ti,ab OR nutri*:ti,ab OR 'physical activ*':ti,ab OR exercis*:ti,ab OR sleep:ti,ab OR stress:ti,ab OR resilience:ti,ab OR smok*:ti,ab OR 'substance use':ti,ab OR alcohol:ti,ab OR lifestyle:ti,ab OR 'combined lifestyle':ti,ab OR 'multicomponent lifestyle':ti,ab OR 'multiple lifestyle':ti,ab) |
| EBSCOhost/CINAHL | Jan–Feb 2024; updated Dec 2025 | ("rheumatoid arthritis" OR MH "Rheumatoid Arthritis") AND (diet OR nutri* OR "physical activ*" OR exercis* OR sleep OR stress OR resilience OR smok* OR "substance use" OR alcohol OR lifestyle OR "combined lifestyle" OR "multicomponent lifestyle" OR "multiple lifestyle") |
| SportsDiscuss | Jan–Feb 2024; updated Dec 2025 | "rheumatoid arthritis" AND (diet OR nutri* OR "physical activ*" OR exercis* OR sleep OR stress OR resilience OR smok* OR "substance use" OR alcohol OR lifestyle OR "combined lifestyle" OR "multicomponent lifestyle" OR "multiple lifestyle") |

**Pubmed**

("Arthritis, Rheumatoid"[MeSH] OR "rheumatoid arthritis"[tiab])

AND

(

diet*[tiab]

OR nutri*[tiab]

OR physical activ* [tiab]

OR exercis*[tiab]

OR sleep* [tiab]

OR stress* [tiab]

OR resilien* [tiab]

OR smok*[tiab]

OR "substance use"[tiab]

OR alcohol* [tiab]

OR lifestyle[tiab]

OR "combined lifestyle"[tiab]

OR "multicomponent lifestyle"[tiab]

OR "multiple lifestyle"[tiab]

)

**Embase**

('rheumatoid arthritis'/exp OR 'rheumatoid arthritis':ti,ab)
AND

diet*:ti,ab

OR nutri*:ti,ab

OR 'physical activ*':ti,ab

OR exercis*:ti,ab

OR sleep*:ti,ab

OR stress*:ti,ab

OR resilien*:ti,ab

OR smok*:ti,ab

OR 'substance use':ti,ab

OR alcohol*:ti,ab

OR lifestyle:ti,ab
OR 'combined lifestyle':ti,ab
OR 'multicomponent lifestyle':ti,ab
OR 'multiple lifestyle':ti,ab

**Cinahl/ebscohost**

(MH "Rheumatoid Arthritis" OR TI "rheumatoid arthritis" OR AB "rheumatoid arthritis")

AND

(

TI (diet* OR nutri* OR physical activ* OR exercis* OR sleep* OR stress* OR resilien* OR smok* OR "substance use" OR alcohol* OR lifestyle OR "combined lifestyle" OR "multicomponent lifestyle" OR "multiple lifestyle")

OR

AB (diet* OR nutri* OR physical activ* OR exercis* OR sleep OR stress OR resilience OR smok* OR "substance use" OR alcohol* OR lifestyle OR "combined lifestyle" OR "multicomponent lifestyle" OR "multiple lifestyle")

)

**Sportdiscus**

(TI "rheumatoid arthritis" OR AB "rheumatoid arthritis")

AND

(

TI (diet* OR nutri* OR physical activ* OR exercis* OR sleep* OR stress* OR resilien* OR smok* OR "substance use" OR alcohol* OR lifestyle OR "combined lifestyle" OR "multicomponent lifestyle" OR "multiple lifestyle")

OR

AB (diet* OR nutri* OR physical activ* OR exercis* OR sleep* OR stress* OR resilien* OR smok* OR "substance use" OR alcohol* OR lifestyle OR "combined lifestyle" OR "multicomponent lifestyle" OR "multiple lifestyle")

)
